# Recommendations for community pharmacy to improve access to medication advice for people from ethnic minority communities: A qualitative person‐centred codesign study

**DOI:** 10.1111/hex.13611

**Published:** 2022-09-26

**Authors:** Anna Robinson, Nicola O'Brien, Laura Sile, Harpreet K. Guraya, Thorrun Govind, Vicki Harris, Guy Pilkington, Adam Todd, Andy Husband

**Affiliations:** ^1^ School of Pharmacy Newcastle University Newcastle upon Tyne UK; ^2^ Population Health Sciences Institute Newcastle University Newcastle upon Tyne UK; ^3^ Department of Psychology Northumbria University Newcastle upon Tyne UK; ^4^ Alumni, School of Pharmacy Liverpool John Moores University Liverpool UK; ^5^ Whale Hill Pharmacy Middlesbrough UK; ^6^ Chair of the English Pharmacy Board Royal Pharmaceutical Society London UK; ^7^ Connected Voice Haref Higham House Newcastle upon Tyne UK; ^8^ West End Family Health Primary Care Network Cruddas Park Surgery Newcastle upon Tyne UK

**Keywords:** codesign, ethnic minority, ethnicity, health inequalities, medication review, qualitative

## Abstract

**Introduction:**

Medicines‐centred consultations are vital to support medicine effectiveness and optimize health outcomes for patients. However, inequalities negatively impact ethnic minority populations when accessing medicines advice. It is important to identify opportunities to improve access for these communities however, knowledge of how best to achieve this is lacking; this study will generate recommendations to improve access to medicines advice from community pharmacies for people from ethnic minority communities.

**Methods:**

A series of codesign workshops, with four groups of patient‐stakeholders, were conducted between September–November 2021; they took place in‐person or via video call (adhering to COVID‐19 restrictions). Existing evidence‐based perceptions affecting access to medicines advice were critiqued and recommendations were generated, by use of reflexive thematic analysis, to improve access for ethnic minority patients. The workshops were audio‐recorded and transcribed verbatim. QSR NVivo (Version 12) facilitated data analysis.

**Results:**

Twelve participants were recruited using purposive sampling; including eight UK citizens, two asylum seekers and two participants in receipt of residency visas. In total, four different ethnic minority groups were represented. Each participant took part in a first and second workshop to share and cocreate recommendations to improve access to medicines advice in community pharmacies. Three recommendations were developed and centred on: (i) delivering and providing culturally competent medicines advice; (ii) building awareness of accessing medicines advice from community pharmacies; and (iii) enabling better discussions with patients from ethnic minority communities.

**Conclusions:**

These recommendations have the potential to support community pharmacy services to overcome ethnic inequalities affecting medicines advice; service commissioners should consider these findings to best meet the needs of ethnic minority patients. Cultural competence training for community pharmacy staff could support the creation of pharmacies as inclusive healthcare settings. Collaborative working with ethnic minority communities could enable specific tailoring of medicines‐centred services to best meet their needs.

**Patient or Public Contribution:**

The National Institute for Health Research (NIHR) and Newcastle University Patient and Public Involvement and Engagement group had extensive input in the study design and conceptualization. Seven patient champions were appointed to the steering group to ensure that the research was conducted, and findings were reported, with cultural competence.

**Trial Registration:**

Not applicable.

## 1 INTRODUCTION

Despite reporting poorer general health when compared to their white counterparts, and being more likely to require medication to manage long‐term illness,^1^ evidence has demonstrated that individuals from ethnic minority backgrounds are less likely to engage in regular consultations to review the appropriateness and effectiveness of their medicines.^2^, ^3^, ^4^ Regular reviews of medication are vital to ensure medicine effectiveness and prescribing safety, thus supporting the optimization of health outcomes for patients.^2^, ^5^, ^6^, ^7^ This study focuses on the provision of medicines‐centred advice for patients from ethnic minority communities, offered by pharmacists and pharmacy teams in the United Kingdom working in a community pharmacy. In the context of this study, medicines advice can include prescription, adherence or compliance reviews; this is rather than structured clinical medication reviews (which require access to clinical information and thus occur more readily in General Practice, Primary Care Networks or secondary care settings) or medication use reviews (which have been discontinued as a community pharmacy service).^8^, ^9^ Medicines advice consultations may also take the form of ad‐hoc interventions made by community pharmacy teams, including the New Medicines Service, or aligned with annual long‐term condition reviews^10^, ^11^ or referrals from other healthcare professionals.^12^, ^13^ These types of medicines advice consultations may differ from those in other countries or healthcare settings, for example, Australian Home Medication Reviews^14^ or Swiss Polymedication Checks.^15^, ^16^ Optimization of patient outcomes is an underpinning goal in the prescribing and provision of medicines however, inequalities affecting access to such advice and support have been previously identified, particularly relating to ethnic minority communities.^17^, ^18^, ^19^

While previous studies have demonstrated the importance of overcoming barriers related to access, specific detail about how best to achieve this is lacking.^4^ Recent work has identified community pharmacies as a setting of strategic importance for the delivery of community‐centred medicines‐related services.^4^, ^20^ Community pharmacists are reported as the most accessible primary care healthcare provider^21^; advantageously, medicines consultations are available without an appointment, often during the evenings and at weekends. Pharmacists have been described as medicines experts with a wealth of knowledge that can support reviews of medications.^22^ However, patients need to be adequately informed on all aspects of a medicine and the associated effects so that they can make an informed decision if, how and when to take it.^3^, ^22^ For ethnic minority communities, this means that medicines information and medicines services must be delivered in the widest sense; in formats and through services that are culturally appropriate and respectful of a person's wishes for instance, around communication, culture and religion.^23^, ^24^

When considering the value that medicines advice consultations can offer in optimizing a person's medication, it is important to (i) better understand existing barriers that may impact those from ethnic minority communities when accessing services and to (ii) identify and explore enablers that may facilitate improved access by these groups. This qualitative co‐designed approach with patients seeks to build greater knowledge and understanding by involving representatives from communities whose needs may remain unmet. Previous work has demonstrated the importance of including the participants' voice within research of this nature.^4^ Through codesign workshops, this study seeks to integrate the voices of those people from ethnic minority populations to gain better insight and create recommendations, on improving access to medicines advice from community pharmacies for people from ethnic minority communities.

## 2 METHOD

### 2.1 Recruitment and sampling

The consolidated criteria for reporting qualitative research (COREQ) checklist was followed for this study (see Supporting Information: File).^25^ This study was conducted during the COVID‐19 pandemic and therefore, UK governmental restrictions were followed throughout. Given the capabilities of digital strategies to support qualitative research, a blended strategy was applied to pragmatically perform and maximize participant recruitment and data collection.

Recruitment was conducted using social media (on the professional Twitter accounts of the researchers, Newcastle University School of Pharmacy, and the Connected Voice charity) and through dissemination to community leaders by charities based in the North of England. All interested participants who contacted the research team were emailed an information sheet and consent form detailing the purpose and aim of the research. Those who expressed an interest in participating in the work were asked to provide written consent and were then enroled in the study. There was no prior relationship established between the researcher and participants before study commencement or recruitment. Inclusion criteria comprised: (i) participants over 18 years of age who were from an ethnic minority group (non‐White British) living in the North of England; (ii) who took one (or more) regular prescription medicine(s); and (iii) who had the capacity to consent to take part in the study. There was no requirement to communicate in the English Language; interpreters were involved throughout the research process for participants that required them. Purposive sampling was used to recruit participants from different ethnic minority groups reflective of the communities living in the North of England, who were of different ages, and who had varying sociodemographic and immigration backgrounds (including UK citizens, those in receipt of residency visas and those who were seeking asylum). Study documentation including the social media advert, participant information sheet and consent form, were translated into different languages to promote inclusivity in the research process (reflecting the languages spoken by the communities residing in the research area, including Bengali, Polish, Punjabi, Mandarin, Romanian and Urdu); all materials were reviewed and approved by the Health Research Authority.

### 2.2 Codesign workshops

**Figure 1:**
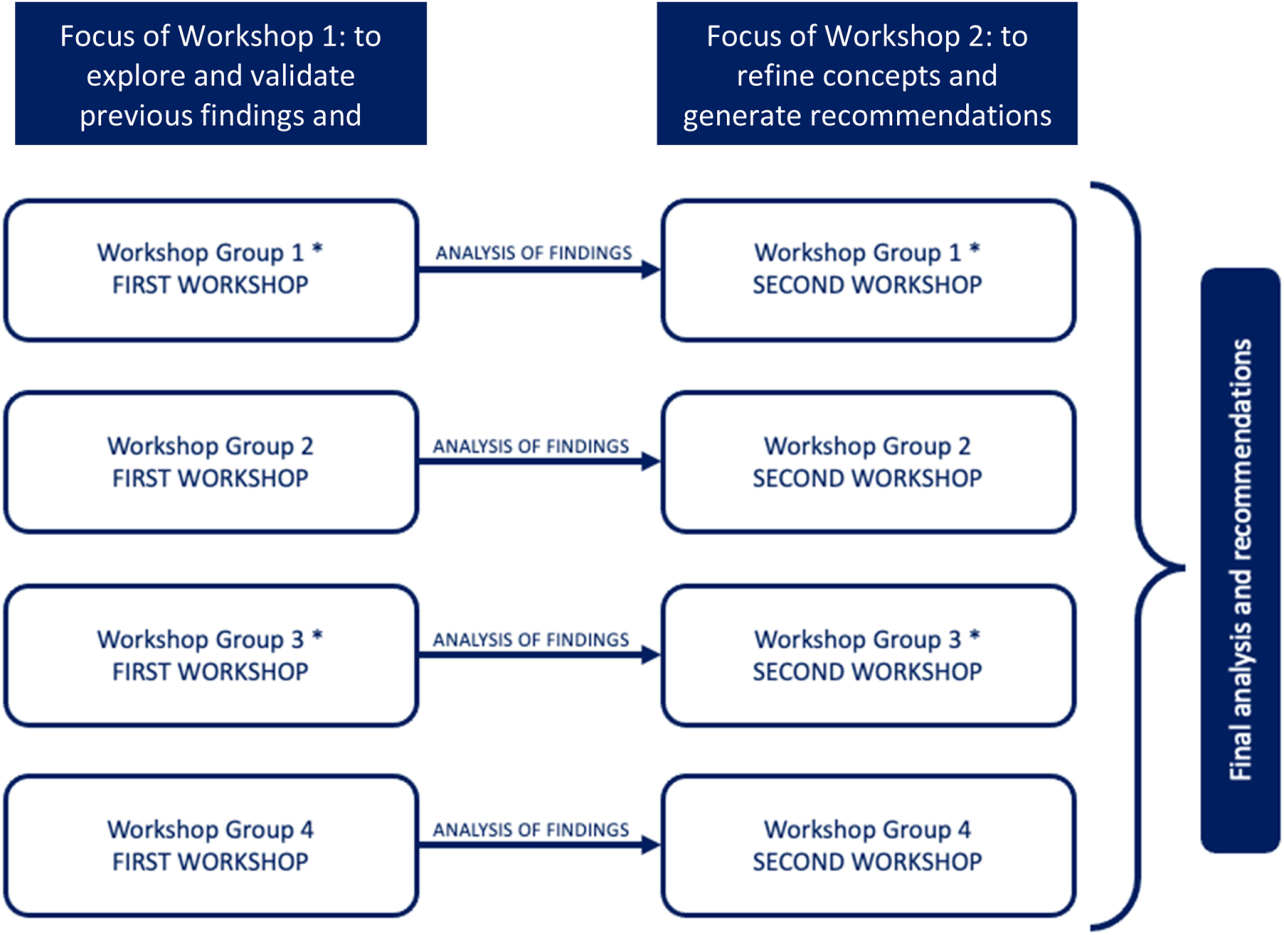
An overview of the codesign workshop structure: demonstrating the first and second workshops for each homogenous group of participants, representative of different ethnic minority communities. N.B. time was taken between workshops to enable the researchers to reflect and make any adaptations from one workshop to the next, as part of the iterative methodology and process of codesign.

Codesign workshops were conducted by two members of the research team (NO a female researcher with expertize in codesign methodology, and AR a female doctoral researcher with experience in qualitative research) between September and November 2021. The codesign workshops were purposely structured and conducted so that the individuals partaking in them were from homogenous ethnic groups^26^; Arabic participants were further split into workshops based on their reported gender. This approach was taken to ensure participant comfort and to appreciate the challenges that may arise between cultural groups and between males and females when discussing health conditions.^27^, ^28^ All participants were offered the choice of which format of the workshop they would prefer (either using Zoom® or in‐person). An interpreter was used to aid discussions in two workshops (Group 1 and Group 3, Figure 1).

There were eight workshops conducted in total; these ran as four pairs of Workshop 1 and Workshop 2. Workshop 1 was designed in such a way as to further explore and validate the findings from two previous qualitative studies by the authors (see Figure 1 and Supporting Information: File).^4^, ^29^ It acted as an opportunity to sense check and assess the face validity of the findings, and for participants to begin identifying core concepts of what constitutes accessibility relating to seeking medicines advice from community pharmacies.^27^, ^28^ Workshop 2 facilitated the refinement of the core concepts and provided the platform for participants to generate recommendations to achieve improvements in access to medicines advice for patients from ethnic minority groups. In particular, the workshop guide explored participant understanding of medicines and taking them safely, seeking advice on what they are prescribed, perceived barriers and facilitators that affect accessing advice about their medicines and lived experiences when accessing such advice from community pharmacy teams. Conducting the workshops and analyzing the data followed an iterative process, during which time was taken for reflection and to make any adaptations from one workshop to the next. This codesign approach will enable the generation of overarching ‘recommendations’ and ‘areas of focus’ that can be adopted as interventions to be applied in community pharmacy settings, with the aim of improving access to medicines advice for people from ethnic minority communities.

### 2.3 Data analysis

All codesign workshops were audio‐recorded to enable data analysis. The audio files were encrypted and transferred electronically (via a password‐protected dropbox) to an external transcription company to be transcribed verbatim; all data were anonymised at the point of transcription. All transcripts were checked for accuracy and correctness by one researcher (A. R.) and participants did not provide comments on the transcripts nor feedback on the results. Any workshops that included an interpreter had their speech (translated to the English language) written in the transcript. The data generated from the first workshop was analysed using a reflexive thematic analysis approach as defined by Braun and Clarke.^30^ This was done for each group separately, such that the design of workshop 2 was based on data from workshop 1; this approach enabled the perspectives from homogenous participant groups to be explored in greater detail. Findings from the second workshop were also analysed using reflexive thematic analysis. Themes were developed following all of the second workshop groups and were refined and named to best reflect participant recommendations to improve access to medicine review services for ethnic minority communities.

The principle of constant comparison guided an iterative process of data collection and analysis.^31^, ^32^ Reflexive thematic analysis was performed by two researchers (A. R. and N. O.); close and detailed reading of the transcripts allowed the two researchers to familiarize themselves with the data. Initial descriptive codes were identified in a systematic manner across the data sets; these were then sorted into common coding patterns, which enabled the development of analytic themes from the data. The themes were reviewed, refined and named as recommendations once coherent and distinctive. Two authors (A. R. and N. O.) performed the data analysis through discussion and, if agreement was not reached, by consensus with members of the wider team (A. H., A. T. and V. H.). Post‐interview field notes enhanced this reflective process. NVivo (version 12) software was used to facilitate data management. Given the research funding timeline and the implications of the COVID‐19 pandemic (further discussed in study limitations), data sufficiency and information power^33^ were reached after conducting four pairs of codesign workshops. To ensure participant confidentiality, patient quotes are attributed to a participant number.

### 2.4 Considerations when reporting participant demographics and ethnicity

Since ethnicity in itself is a multifaceted and changing phenomenon, collecting data on a person's ethnic group is complex. There is no consensus on what constitutes an ethnic group when, often, it is something that is self‐defined and subjective to an individual. Efforts were taken to report a multitude of factors (including a person's first language, religion and citizenship status) to demonstrate the layers that accompany discussions around ethnicity. The National Institutes of Health^34^ and UK Office of National Statistics^35^ guides informed the initial reporting of participant ethnicity for this study. Table 1 includes a column for self‐identified ethnicity; this has been reported verbatim for each study participant.

**Table 1:** Participant characteristics and codesign workshop formats

Codesign workshop group	Participant no.	Sex (M/F)	Age (years)	Ethnicity (as per Office for National Statistics guidance for ethnicity reporting)^35^	Ethnicity (self‐identified by participant, reported verbatim)	Workshop format	Interpreter required (Y/N)	First language	Time living in England	Religion	Citizenship status at the time of the workshop	First and second workshop duration (min)	
Group 1	1	F	33	Other ethnic group	‘Arab ethnicity’	Video call	N	Arabic	3 years	Muslim	Residency visa	110	95
	2	F	34	Other ethnic group	‘Arab’	Video call	N	Arabic	3 years	Muslim	Residency visa		
	3	F	38	Other ethnic group	‘Arab’	Video call	Y	Arabic	3 years	Muslim	Asylum seeker		
	4	F	39	Mixed or multiple ethnic groups	‘Mixed – Arab and Turkish’	Video call	N	Arabic	8 years	Muslim	Asylum seeker		
Group 2	5	M	35	Asian or Asian British	‘British Asian Pakistani’	Video call	N	Urdu	35 years	Atheist	UK Citizen	129	118
	6	M	55	Mixed or multiple ethnic groups	‘Mixed British and Arab’	Video call	N	English	41 years	Atheist	UK Citizen		
Group 3	7	F	55	Asian or Asian British	‘Punjabi Indian’	In‐person	N	Punjabi	29 years	Sikh	UK Citizen	132	127
	8	M	72	Asian or Asian British	‘British Indian’	In‐person	N	Punjabi	54 years	Sikh	UK Citizen		
	9	F	52	Asian or Asian British	‘Indian’	In‐person	Y	Punjabi	21 years	Sikh	UK Citizen		
Group 4	10	M	65	White	‘Israeli’	In‐person	N	English	65 years	Jewish	UK Citizen	103	77
	11	F	42	White	‘White British’	In‐person	N	English	35 years	Jewish	UK Citizen		
	12	F	60	White	‘British Jewish’	In‐person	N	English	60 years	Jewish	UK Citizen		

Abbreviations: F, female; M, male; N, no; Y, yes.

## 3 RESULTS

### 3.1 Participant demographics

Twelve participants in total were recruited and took part in the four pairs of codesign workshops for this study; each group of participants took part in a first and second workshop to share their perspectives and make recommendations on improving access to seeking medicines advice from community pharmacies for people from ethnic minority communities. Of the 12 participants, there were eight United Kingdom citizens, two people in receipt of residency visas and two asylum seekers. There were no refusals to partake and there were no participant dropouts throughout the course of the study. The average age of participants was 48 years (SD: 12.68) and four different ethnic groups were represented within the sample. All workshops were conducted in the English language however, two participants required an interpreter to aid in discussions (who provided interpretation from Urdu and Punjabi to the English language). Two sets of codesign workshops were conducted in person and two sets were performed using video call software, Zoom®. The average duration of workshop 1 was 119 min (SD: 12.3) and the average duration of workshop 2 was 104 min (SD: 19.6).

**Figure 2:**
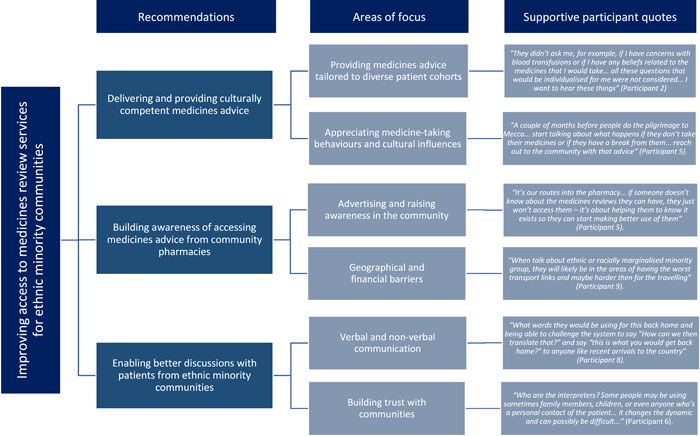
Improving access to medicines advice from community pharmacies for people from ethnic minority communities: detailing the three high‐level recommendations and six areas of focus developed from this codesign study, accompanied by illustrative verbatim quotes from participants to support the recommendations.

Findings from this study have identified three high‐level recommendations that impact access to medicines advice by people from ethnic minority communities: (i) delivering and providing culturally competent medicines advice, (ii) building awareness of accessing medicines advice from community pharmacies, and (iii) enabling better discussions with patients from ethnic minority communities. Across these recommendations, six areas of focus were identified as strategies that could support improved access to, and provision of, medicines advice for people from ethnic minority communities. These areas of focus included providing medicines advice tailored to diverse patient cohorts; appreciating medicine‐taking behaviours and cultural influences; advertising and raising awareness in the community; geographical and financial barriers; verbal and nonverbal communication; and building trust with communities (as demonstrated in Figure 2).

### 3.2 Recommendation 1: Delivering and providing culturally competent medicines advise

In all workshops, participants discussed the importance of tailoring medicines advice so that healthcare professionals recognized each individual and their needs. Participants acknowledged that different cultural and religious behaviours existed within the diverse cohorts of people from ethnic minority communities accessing pharmacies; consequently, these behaviours may influence a person's decision or ability to take their medicines. By better tailoring medicines advice provided to appreciate this, barriers to medicines advice seeking could be overcome for members of ethnic minority communities.

#### 3.2.1 Area of focus 1: Providing medicines advice tailored to diverse patient cohorts

One participant drew on their previous experiences to highlight the gap that could be addressed by better tailoring and individualization of medicines advice provision. They reported how their personal preferences about treatment options (including medicines) were not discussed in their consultations, which led to feelings of ‘them not caring about me as a person… to me, these things need considered because it affects me as a person’ (Participant 2). They explained how ‘my own preferences… these should be the things pharmacists and doctors consider for us (Arabic community) but maybe they don't know them, maybe they aren't taught them, but there's a simple way of asking me to find this stuff out’ (Participant 2).

Participants discussed enablers which could support the provision of medicines advice in a culturally competent manner, many of which related to the education and training of pharmacy staff. Specifically, ‘training for (pharmacy teams) around other cultures, or cultures different to their own so they can be more aware’ (Participant 5), ‘more learning about the people's culture who live in the area around (the pharmacy)’ (Participant 6) and ‘speaking to me, speaking to my neighbours, asking us Muslims because we can tell to them if it is something they do not know about’ (Participant 2). It appeared that cultural competence was essential in the education of pharmacy staff–both in relation to a person's existing prescribed medications and in supporting decision‐making for future treatment. Two participants discussed that upon prescribing any medication and providing medicines advice, the healthcare professional should have consulted about, and considered their culture and beliefs; their rationale for this was to understand whether there were any excipients in the medicine that may be unsuitable for their consumption.

They didn't ask me, for example, if I have concerns with blood transfusions or if I have any beliefs related to the medicines that I would take (Participant 2).

#### 3.2.2 Area of focus 2: Appreciating medicine‐taking behaviours and cultural influences

Participants discussed that the provision of medicines advice should place emphasis upon understanding a person's medicine‐taking behaviour and their adherence to medicines. Many participants described that their own medicines‐taking followed and respected the religious behaviours of their community; one Sikh participant discussed how they did not take capsule‐based medications as ‘it could be like gelatine form, where the Indians are not supposed to take any material from cows or beef, it's a religious barrier for us’ (Participant 7). One Muslim participant also reported how they needed to check ‘does the medicine contain pork, does it have a gelatine capsule and does it contain alcohol? There's all of these different barriers, culturally and religiously, to make sure that people are looked after in a way that they are wanting to follow’ (Participant 5).

All four workshop groups discussed the ‘impact of times in religious calendars that medicines services should be aware of, like Ramadan and times of fasting’ (Participant 4). Each workshop group identified that, when providing medicines advice, community pharmacists could proactively discuss ‘these topics with members of the community that use their pharmacies… start having those conversations to hear how the medicines support can be given in respect of a person's choice to fast or do a pilgrimage’ (Participant 6). It was imperative that medicines advice was delivered in a culturally competent manner that respected and acknowledged the views of individuals, as well as their community as a whole.

### 3.3 Recommendation 2: Building awareness of accessing medicines advice from community pharmacies

The workshop participants highlighted the importance of building awareness of accessing medicines advice from community pharmacies. Challenges were discussed around not knowing that medicines advice could be obtained from community pharmacies, as well as understanding the rationale of seeking such advice and acknowledging the physical and geographical barriers affecting a person accessing such advice; these were perceived as the main factors affecting rates of access and engagement.

#### 3.3.1 Area of focus 1: Advertising and raising awareness in the community

Two participants reported not knowing about the possibility of seeking medicines advice from community pharmacies before arriving in the UK. One explained ‘when I coming here from India, no such thing existed that I knew of in my home country… (but) we learn about asking the pharmacist when we came here to England’ (Participant 7). Participants spoke of others in their community who they believed would likely be the same as them and discussed potential recommendations to ‘improve the people's knowing about (this), especially when they first come to this island… they maybe don't know these things… it is important to help them know and learn because it is for their benefit’ (Participant 9).

Another participant described how this potential gap in knowledge may hinder someone's engagement with the service as ‘they might think “why do I have to go through my medical history with you?” but they might not understand the purpose of what it is for’ (Participant 5). The same participant described how raising awareness of seeking medicines advice from community pharmacies, through advertising, could help to establish wider community‐level knowledge. Community‐wide advertising could help ‘get the message out to the new arrivals… and also for people who maybe living here long time in UK but maybe not sure of’ the purpose, rationale and benefits of seeking medicines advice (Participant 9). Community pharmacies were discussed as being ‘at the heart of communities and we need to make better use of them to support people from minority communities’ (Participant 5). Participants described forms of advertising that community pharmacies could employ to educate members of community groups, including ‘advertising the medicines review in the local shops, like my local Halal food store, where you'd get the footfall from the people who might benefit the most’ (Participant 5), and ‘the local Asian radio station even, they might spread the word about (medicine) reviews if you were the local (pharmacy)’ (Participant 6). In addition, participants discussed how trusted members of the community, such as religious leaders in their community, could assist in raising awareness of such services. One participant described ‘put posters up in the Mosque, they'll see them regularly… read them and probably talk about them with other people attending’ (Participant 6).

Hearing about it from people they trust… like the Imam, or equivalent if they aren't Muslim, that's probably going to be someone in their community they listen to and respect (Participant 5).

#### 3.3.2 Area of focus 2: Geographical and financial barriers

Physical access to a community pharmacy, as a place to discuss and review medication with a community pharmacist, was described as ‘a space, a safe space, where everyone should know they can go to speak about their tablets’ (Participant 6). However, many reported that there may be potential challenges based around access for people from ethnic minority communities and, in particular, new arrival refugees. One participant described the importance of people having ‘awareness on where (the community pharmacy) is, how to get there, which bus to use, those types of things’ (Participant 1). Another participant described financial barriers associated with travelling which their relatives recently experienced, ‘even negotiating which bus to take to get there, which certainly isn't easy if you're new to the country, don't speak the language, and only receiving minimal (monetary) benefits’ (Participant 6).

### 3.4 Recommendation 3: Enabling better discussions with patients from ethnic minority communities

Participants acknowledged the role that community pharmacists can play in enabling discussions with different ethnic minority communities living in the local area. Both verbal and nonverbal communication methods were perceived to support relationship‐ and trust‐building between the healthcare professional team and members of the communities.

#### 3.4.1 Area of focus 1: Verbal and nonverbal communication

Many workshop participants discussed the immediate barriers that can be faced when a person ‘does or does not speak English’ (Participant 6). At a minimum, participants recognized that, from a patient safety perspective, it was regarded as ‘vital that a person understands what (medications) they're taking and why… they can't even speak English so how do you best get that message about medicines across to them?’ (Participant 5).

Medicines‐specific instructions were considered a significant area where verbal communication could be improved between pharmacy professionals and patients. One participant described experiences of supporting an older relative to take their medication and ‘even explaining to them how to actually take it, how to take the tablet, like “swallow with a glass of water, not a cup of tea”—that's an instruction that's really important to make sure the medicine works properly, but no one had explained that to her’ (Participant 6). Supporting a person to understand any differences and equivalencies in the medicines prescribed in the UK compared to their home country was also deemed important as a point of education and reassurance.

What words would they be using for this back home and being able… (to) say ‘this is what you would get back home’ to anyone like recent arrivals to the country (Participant 8).

The role of written translations, ‘writing out the instructions for them’ and ‘including the specific medicines' details on the label or something, like when in the day you take it or if you can have it with a cuppa’ were raised as strategies to support communication (Participant 5). However, one participant believed that instructions ‘should be written in English… otherwise there becomes no incentive to learn English if it is always for you in Punjabi’ (Participant 7). There was also discussion around written translations not being suitable for all languages, in particular those which are primarily spoken and do not have a written form, including variations of Chinese.

The value of interpreter services to support person‐centred consultations was discussed; however, participants commented that the interpreter services did not appear to extend to community pharmacies to support in pharmacists providing medicines advice, compared with consultations in other healthcare settings. One participant questioned ‘how the interpreter can be with the GP for his appointment, but why not with pharmacist for his?’ (Participant 8). It appeared that the context of booking appointments in advance may facilitate better discussions in pharmacy settings, as ‘when I need translator for GP appointment, I always get told to have appointment the next day so they can organise someone to speak Punjabi with me. But (in the pharmacy), they cannot do this as it is more walk‐in not normally booked day or time… maybe it is possible to do the same?’ (Participant 9). One participant discussed acting as an interpreter for a neighbour, but highlighted the drawbacks of doing so when it concerned privacy and health matters; the use of external interpreter services was preferred over using family members or friends.

#### 3.4.2 Area of focus 2: Building trust with communities

Another factor influential in enabling better discussions with ethnic minority communities stemmed from building relationships of trust between communities and healthcare professionals. A member of the Jewish community discussed the relationship built with their pharmacist Rabbi and described how ‘members of our community know they can go and ask him anything… he is a trusted and respected figure in our community… it is important to us to know we can trust him’ (Participant 11). This was a common theme discussed by participants from other ethnic groups too. Building trust was perceived to be an essential two‐way exchange; one participant discussed the importance of healthcare professionals making the initial first steps to capitalize on opportunities and ‘build that credibility amongst the communities, to show that expertise and open the dialogue’ (Participant 6).

The employment of staff within the healthcare professional team, who were reflective of the ethnic minority community, was acknowledged as another facilitator in building trust. Participants described feelings of reassurance and ease when they were able to identify a member of staff they could easily communicate with—this appeared to be a feeling of comfort both in a practical sense of sharing the same language and ‘having someone they could confide in by speaking their own language and removing that stress of not knowing how to say, how to communicate’ (Participant 6), but also in a cultural sense where they knew cultural understanding would be shared between them and the healthcare professional. One participant described ‘I said to (pharmacist) “for Ramadan” and she knew exactly what my worry was because she was a Muslim too’ (Participant 1). Integrating staff from ethnic minority groups into the pharmacy team could also facilitate better access and engagement from local communities. One participant described feeling reassured that ‘I could go back in and ask any question about my medicines if (the member of staff) was working there’ and discussed how they would ‘ask them if they can explain me in my language and then I feel comfortable to ask anything’ (Participant 7).

## 4 DISCUSSION

This study adds to the growing evidence base considering access to medicines‐specific advice for ethnic minority populations.^2^, ^3^, ^4^, ^22^, ^29^ This codesign research provided a platform to share the voices of members from ethnic minority communities and generate person‐centred recommendations to improve access to seeking medicines advice from community pharmacies. The three recommendations generated from this codesign study may act as high‐level starting points for interventions, and should now be considered by community pharmacy service commissioners to better meet the needs of ethnic minority patient cohorts. Future follow‐up research should be done to provide insight into whether these recommendations and strategies for improvement can generate meaningful impacts, both from the perspective of patients and the pharmacy professionals involved. Further research is also required to identify whether interventions formed by these recommendations are appropriate and relevant for all ethnic minority groups, or whether specific communities may benefit from additional, tailored or alternative approaches to best meet their needs.

The importance of delivering individualized and culturally competent medicines‐focused advice was highlighted.^29^, ^36^ Appreciation of cultural and religious beliefs was acknowledged as facilitators that may improve the provision of accessible and personalized medicines services. Specifically in this study, participants highlighted the importance of shared conversations with pharmacy professionals around medication excipients, alcohol‐content and religious festivals that affect adherence to medications (including periods of fasting). Perspectives raised in the workshops echoed those in the wider literature, where a person's medicines‐taking behaviour and subsequent access to medicines services can be influenced by their culture.^37^, ^38^, ^39^, ^40^ To enable culturally appropriate conversations and support the delivery of culturally competent medicines consultations, participants recognized the need to underpin community pharmacy service design and delivery with improved staff training. Knowledge gaps around what cultural competence training is required to support the education of qualified pharmacists and pharmacy students have previously been identified,^41^ and questions have been raised about the format, content and optimization of this training too.^42^, ^43^, ^44^, ^45^, ^46^ In the updated standards for the initial education and training of pharmacists, the General Pharmaceutical Council placed emphasis on equality, diversity and inclusion to address health inequalities.^47^ Future work should address how best to deliver this and further investigate the optimal content of cultural competence training programmes for pharmacists and members of the pharmacy team, to meet the needs of the ethnic minority communities accessing medicines advice.

Participants recognized the supportive role that community pharmacists could play in establishing connections with ethnic minority communities; in doing so, barriers affecting access could be overcome through greater community awareness and relationship‐building.^20^, ^21^ The availability of community pharmacists and pharmacy staff could provide an opportunity for fostering trusting, long‐term relationships with patients and their families.^48^ Parallels could be drawn to findings in the wider literature where tailored approaches have been implemented in community pharmacy services to best build relationships and improve the patient experience with specific patient cohorts; these have included community pharmacy medication services for people with diabetes,^49^ mental health disorders,^50^ epilepsy,^51^, ^52^ and in the delivery of addiction^53^ and contraceptive clinics.^54^ Future work should seek to explore in greater depth through close, collaborative working with individual ethnic minority communities. In doing so, this may enable greater insight into specific tailored approaches or mechanisms that best meet the needs of individual underserved populations.^29^

Interpreter services were acknowledged as a possible mechanism to support improved access to seeking medicines advice; however, participants recognized the contrast in availability and prevalence of interpreter services in community pharmacies compared to other UK healthcare settings, like General Practice surgeries. This echoes findings from other high‐income countries where community pharmacists have played a role in delivering medicines reviews for patients from ethnic minority communities, including in Australia,^46^, ^55^, ^56^ the United States,^57^, ^58^ Japan,^59^ New Zealand^60^ and Denmark.^61^ Given the findings from this study, researchers, service commissioners and service designers should next look at mechanisms to better facilitate the provision of interpreter services within community pharmacies. The employment of staff from ethnic minority communities was acknowledged as a potential twofold facilitator; both to support improved access to community pharmacies, and overcome the lack of interpreter presence to enable patient‐centred consultations. This finding supports the overarching principles of good practice for community pharmacy teams, to address health inequalities (point 1.2.6) in the National Institute of Health and Care Excellence Guidance.^62^ Wider studies based on education have acknowledged the relationship‐building that can result between teaching staff and students from the same ethnic minority communities^63^, ^64^, ^65^; where terminology such as ‘belongingness’ and ‘co‐identification’ has been used to describe bridging gaps and creating more inclusive environments. Health‐related parallels may exist that could support the creation of pharmacies as inclusive health‐seeking environments for members of the community.^23^, ^46^, ^62^ However, the authors postulate that this approach may only pose as a single‐pronged solution to the growing communication barriers faced amongst an increasingly diverse patient population in the United Kingdom; whereby additional approaches may be needed, depending on the needs of the populations living locally to the community pharmacy.

Guidance from the NIHR INCLUDE project and the National Institute for Health Research (NIHR) toolkit for “Increasing participation of Black, Asian and Minority Ethnic (BAME) groups in health and social care research” supported the sampling, recruitment and conduct of this study.^26^, ^66^ Despite the associations between healthcare accessibility and ethnic inequalities, people from ethnic minority communities continue to remain underrepresented participants in health and social care research.^3^, ^67^ This study aimed to include the voices of these participant groups, which is a key strength. Efforts were taken to involve participants of different ages (ranging from 33 to 72 years), from asylum‐seeking groups (*n* = 2) and those in receipt of a residency visa (*n* = 2). The authors recognize, however, that the participant sample was limited in representation from some ethnic minority communities residing in the North of England; for example, from African‐Caribbean ethnic groups. Although workshops were conducted with an interpreter presence to minimize barriers in communication for those who did not speak English fluently, the authors recognize that running workshops in languages other than English may have encouraged wider participation; future studies may wish to employ this approach to complement codesign workshops conducted in the English language. The intended method of in‐person data collection for the codesign workshops was impacted by the COVID‐19 pandemic; although 2 sets of workshops took place in person, the remaining 2 sets were conducted remotely. Remote interview techniques have been praised for assisting with the continuation of qualitative research during the pandemic.^29^, ^68^, ^69^, ^70^, ^71^ The authors acknowledge that the codesign workshop groups were ethnically‐homogenous, however, this decision was based on cultural competence training and done to enable discussions in a safe environment for participants, whilst also respecting the cultural practices and beliefs of communities. This study focused on ethnic minority populations living in the North of England, meaning that findings may not be generalizable to those of other countries; however, the high‐level recommendations generated in this study could, and should, be adopted to overcome barriers for people from ethnic minority communities worldwide. This study uses codesign with patient stakeholders and therefore, before proposed recommendations are used to inform the design and implementation of an improved medication review service, there is a need to also explore the perspectives of additional stakeholder groups.

### 4.1 Researcher positionality and reflexivity statement

When conducting research on ethnicity, it is important to acknowledge the positionality and reflexivity of the research team. Authors A. R., N. O., V. H., G. P., A. T. and A. H. recognized their privilege as nonethnic minority UK citizens. Authors T. G., H. K. G. and L. S. were appointed as patient champions in this study team to represent the ethnic minority communities involved in the study; these authors ensured cultural appropriateness and sensitivity throughout the entire research process.

## 5 CONCLUSION

This study used a codesign approach with patient stakeholders to identify opportunities and generate recommendations to improve access to seeking medicines advice for people from ethnic minority communities; these centred on: (i) delivering and providing culturally competent medicines advice; (ii) building awareness of accessing medicines advice from community pharmacies; and (iii) enabling better discussions with patients from ethnic minority communities. The results have important implications for overcoming ethnic inequalities in access to medicines advice; steps should be taken to address knowledge gaps around cultural competence training of pharmacy staff to enable the creation of community pharmacies as inclusive healthcare settings. In addition, enabling person‐centred discussions was deemed significant and methods for improving communication within pharmacies should be further explored. Close, collaborative working with individual ethnic minority communities could enable specific tailoring of the design and delivery of medicines advice services that best meet the needs of the ethnic minority communities.

## AUTHOR CONTRIBUTIONS

Anna Robinson was appointed Research Assistant for this project and led on the data collection and writing of this manuscript. Andy Husband and Adam Todd oversaw the running of this project as Principal Investigators and provided project management expertize. Nicola O'Brien provided qualitative methodological input and codesign expertize. Guy Pilkington and Vicki Harris supported the recruitment of participants and links to ethnic minority communities. Thorrun Govind, Harpreet K. Guraya and Laura Sile contributed in their appointment as patient champions and ensured cultural appropriateness and sensitivity throughout the research process. All authors read, provided comments on, and approved the final manuscript.

## CONFLICT OF INTEREST

The authors declare no conflict of interest.

## ETHICS STATEMENT

Ethical approval was obtained from the NHS Health Research Authority (HRA) and Care Research Wales (reference: 21/HRA/1426).

## Supporting information

Supporting information.

## Data Availability

The data that support the findings of this study are available from the corresponding author upon reasonable request.
