# Supplementary material for: Recommendations for community pharmacy to improve access to medication advice for people from ethnic minority communities: A qualitative person‐centred codesign study
Source: Health Expect. 2022 Sep 26;25(6):3040–52. doi: 10.1111/hex.13611 (PMC9700185; doi:10.1111/hex.13611)
Supplement: Supplementary file 1 — Supporting information. [file HEX-25--s001.docx]

**Item 1:**

**COnsolidated criteria for REporting Qualitative studies (COREQ): 32-item checklist.**

| **Number** | **Item** | **Guide questions / description** | **Reported on manuscript page** |
| --- | --- | --- | --- |
| **Domain 1: research team and reflexivity** | | | |
| **Personal characteristics** | | | |
| 1 | Interviewer | Which author(s) conducted the interviews? | 7 |
| 2 | Credentials | What were the researcher’s credentials? *E.g., PhD, MD* | 7 |
| 3 | Occupation | What was their occupation at the time of the study? | 7 |
| 4 | Gender | Was the researcher male or female? | 7 |
| 5 | Experience and training | What experience or training did the researcher have? | 7 |
| **Relationship with participants** | | | |
| 6 | Relationship established | Was a relationship established prior to study commencement? | 6 |
| 7 | Participant knowledge of interviewer | What did the participants know about the researcher?  *E.g., reason for doing the research* | 6 |
| 8 | Interviewer characteristics | What characteristics were reported about the interviewer?  *E.g., bias, assumptions, reasons and interests in the research topic* | 6 + 7 + 22 |
| **Domain 2: study design** | | | |
| **Theoretical framework** | | | |
| 9 | Methodological orientation and theory | What methodological orientation was stated to underpin the study?  *E.g., grounded theory, ethnography, discourse analysis* | 7 + 8 |
| **Participant selection** | | | |
| 10 | Sampling | How were participants selected? *E.g., purposive, convenience, consecutive* | 6 |
| 11 | Method of approach | How were participants approached? *E.g., face-to-face, telephone, email* | 6 |
| 12 | Sample size | How many participants were in the study? | 10 |
| 13 | Non-participation | How many people refused to participate or dropped out (with reasons)? | 10 |
| **Setting** | | | |
| 14 | Setting of data collection | How was the data collected? *E.g., home, clinic, workplace* | 7 + 8 |
| 15 | Presence of non-participants | Was anyone else present besides the participant and researcher? | 7 + 8 |
| 16 | Description of sample | What are the important characteristics of the sample? *E.g., demographic data* | 10 |
| **Data collection** | | | |
| 17 | Interview guide | Were questions and prompts provided by the authors? | Supplementary file (item 2) |
| 18 | Repeat interviews | Were repeat interviews carried out? If yes, how many? | 7 + 8 |
| 19 | Audio/visual recording | Did the researcher use audio or visual recording to collect the data? | 8 |
| 20 | Field notes | Were field notes made during/after the interview? | 8 |
| 21 | Duration | What was the duration of the interviews? | 10 + Table 1 |
| 22 | Data saturation | Was data saturation discussed? | 9 |
| 23 | Transcripts returned | Were transcripts returned to participants for comment/correction? | 8 |
| **Domain 3: analysis and findings** | | | |
| **Data analysis** | | | |
| 24 | Number of data coders | How many data coders coded the data? | 8 |
| 25 | Description of the coding tree | Did authors provide a description of the coding tree? | Not applicable |
| 26 | Derivation of themes | Were themes identified in advance or derived from the data? | Not applicable |
| 27 | Software | What software, if applicable, was used to manage the data? | 8 + 9 |
| 28 | Participant checking | Did participants provide feedback on the findings? | 8 + 9 |
| **Reporting** | | | |
| 29 | Quotations presented | Were participant quotations presented to illustrate the themes / findings? Was each quotation identified? E*.g., participant number* | 11-18 |
| 30 | Data and findings consistent | Was there consistency between the data presented and the findings? | Yes |
| 31 | Clarity of major themes | Were major themes clearly presented in the findings? | Yes |
| 32 | Clarity of minor themes | Is there a description of diverse cases or discussion of minor themes? | Yes |

**Item 2:**

1. **Co-design workshop schedule**

Researcher NO (qualitative research and co-design methodological expertise) will conduct the co-design workshops, with Researcher AR (Research Assistant with qualitative research expertise) acting as facilitator. It is estimated that each co-design workshop will take approximately 1-2 hours.

Prior to the workshop beginning, the researchers will establish ground rules for the session to protect the dignity and confidentiality of the participants. The participants will be reminded that the workshops will be audio-recorded so that the discussions (and data) can be transcribed for analysis. Once the workshop recording has been transcribed and anonymised, the recording will be deleted. Anonymisation of the transcript will ensure that the participants cannot be identified.

The workshop will begin with introductions, a brief reminder of the participant information sheet and what the study entails, and then follow a semi-structured flow. In the first workshop, we will use the main themes identified in the Phase 1 interviews as prompts for discussion (see Figure 1 below). The first workshop will act as an opportunity to ‘sense check’ and validate the findings, whilst working towards development of recommendations (using participant opinions). The data that comes from this workshop will be analysed by the research team and used to shape the format of workshop 2.

The second workshop will be used to develop the themes and core recommendations, and identify the processes and support mechanisms needed to facilitate improved access to pharmacy medicine reviews.

1. **Co-design workshop topic guide**

The workshop 1 questions will be based around the following topics:

1. (Broader) Experiences of engaging with health in general / GP / pharmacy service
2. (Broad) Experiences of taking medicines
3. (Narrowing) Experiences of engaging with medicines review
4. (Narrow) Participant understanding of medicines review
5. (Focused) Access to medicines review
6. (Focused) Barriers to accessing medicines reviews
7. (Focused) Facilitators to improve access to medicines reviews
8. (Focused) Delivery of medicines reviews

Workshop 2 questions will be informed by the findings from workshop 1.
